# Inosine Released from Dying or Dead Cells Stimulates Cell Proliferation *via* Adenosine Receptors

**DOI:** 10.3389/fimmu.2017.00504

**Published:** 2017-04-27

**Authors:** Jin Chen, Ricardo A. Chaurio, Christian Maueröder, Anja Derer, Manfred Rauh, Andriy Kost, Yi Liu, Xianming Mo, Axel Hueber, Rostyslav Bilyy, Martin Herrmann, Yi Zhao, Luis E. Muñoz

**Affiliations:** ^1^Department of Rheumatology and Immunology, West China Hospital, Sichuan University, Chengdu, China; ^2^Department of Internal Medicine 3 – Rheumatology and Immunology, Friedrich-Alexander-University Erlangen-Nürnberg (FAU), Universitätsklinikum Erlangen, Erlangen, Germany; ^3^Department of Radiation Oncology, Friedrich-Alexander-University Erlangen-Nürnberg (FAU), Universitätsklinikum Erlangen, Erlangen, Germany; ^4^Kinder- und Jugendklinik, Universitätsklinikum Erlangen, Erlangen, Germany; ^5^Danylo Halytsky Lviv National Medical University, Lviv, Ukraine; ^6^Laboratory of Stem Cell Biology, State Key Laboratory of Biotherapy, West China Hospital, Sichuan University, Collaborative Innovation Center for Biotherapy, Chengdu, China

**Keywords:** melanoma, adenosine receptor, inosine, proliferation, repopulation, apoptosis, necrosis

## Abstract

**Introduction:**

Many antitumor therapies induce apoptotic cell death in order to cause tumor regression. Paradoxically, apoptotic cells are also known to promote wound healing, cell proliferation, and tumor cell repopulation in multicellular organisms. We aimed to characterize the nature of the regenerative signals concentrated in the micromilieu of dead and dying cells.

**Methods:**

Cultures of viable melanoma B16F10 cells, mouse fibroblasts, and primary human fibroblast-like synoviocytes (FLS) in the presence of dead and dying cells, their supernatants (SNs), or purified agonists and antagonists were used to evaluate the stimulation of proliferation. Viable cell quantification was performed by either flow cytometry of harvested cells or by crystal violet staining of adherent cells. High-performance liquid chromatography and liquid chromatography coupled with mass spectrometry of cell SNs were deployed to identify the nature of growth-promoting factors. Coimplantation of living cells in the presence of SNs collected from dead and dying cells and specific agonists was used to evaluate tumor growth *in vivo*.

**Results:**

The stimulation of proliferation of few surviving cells by bystander dead cells was confirmed for melanoma cells, mouse fibroblasts, and primary FLS. We found that small soluble molecules present in the protein-free fraction of SNs of dead and dying cells were responsible for the promotion of proliferation. The nucleoside inosine released by dead and dying cells acting *via* adenosine receptors was identified as putative inducer of proliferation of surviving tumor cells after irradiation and heat treatment.

**Conclusion:**

Inosine released by dead and dying cells mediates tumor cell proliferation *via* purinergic receptors. Therapeutic strategies surmounting this pathway may help to reduce the rate of recurrence after radio- and chemotherapy.

## Introduction

In cancer therapy, most of the treatments aim to induce death of the malignant cells. Apoptosis has been reported as an ongoing mechanism during cancer treatment (1), and necrosis is also a common finding after various types of antineoplastic actions (2, 3). Causing massive cell death in solid tumors changes dramatically the tumor microenvironment and triggers biological reactions in the host and tumor (4). In multicellular organisms, dying cells require swift recognition and efficient removal by a process known as clearance of apoptotic cells (5). This event is habitually coupled with regeneration and healing processes and contributes to the restitution of the damaged organ. Before the clearance of apoptotic cells begins, dying cells release a plethora of “find me” signals that facilitate their prompt removal by phagocytes (6). Among “find me” signals reported to date count the nucleotides ATP and UTP (7), lysophosphatidylcholine (8), fractalkine (CX3CL1) (9), and sphingosine 1-phosphate (10).

Apoptosis also plays a pivotal role in tumor cell repopulation (11) and in demodulation of antitumor responses (12, 13). Animals inoculated with a mixture of viable and irradiated (X-rays) tumor cells exhibited, already over 50 years ago, a reduction of the survival time and an enhancement of tumor growth (14). More recently, several apoptosis-related regeneration phenomena have been reported. β-Catenin-directed Wnt signaling is involved in the compensatory proliferation after apoptotic stimuli in lower multicellular organisms (15). Stroma cells derived from human cancer promote the tumor progression after radiotherapy by the paracrine action of another member of the Wnt family, Wnt16B (16). The most convincing evidence so far includes PGE2 released by dying tumor cells in a caspase 3-dependent manner as a potent growth-stimulating factor that may support tumor repopulation after radiotherapy (17). Our studies showing the growth of melanoma cells by irradiated homologous cells confirm that apoptosis is involved in the compensatory proliferation of neighboring surviving cells (18). However, the role of further “find me” signals released by tumor cells killed by chemo- or radiotherapy in the repopulation of tumors is controversial and needs to be clarified.

All these mediators are expected, in the first place, to establish a gradient for phagocyte attraction (6). However, some of them lack stability to reach long-range targets and their effects may be more important locally on surviving sister cells. Nucleotides are unlikely to serve as long-range “find me” signals to phagocytes since they are readily degraded by extracellular nucleotidases (19). The most important ectonucleotidases CD73 and CD39 are expressed ubiquitously in the human organism and especially on tumor cells (20, 21). Nucleotides are, therefore, considered the main source of extracellular nucleosides in the tumor microenvironment and the concentrations of adenosine range from 0.2 to 2.4 μM (22). The effects of the second messenger adenosine are pleiotropic and widespread (23) and have been associated with the promotion of tumor growth but until now indirectly through its effects on the adaptive immune system (24).

Since B16F10 melanoma cells readily respond with enhanced proliferation in the absence of immune cells *in vitro* when they are stimulated with dead and dying homologous cells (18), we aimed to identify the factors produced by dead and dying cells responsible for this effect. First, we ascertained that the factor promoting proliferation is a non-proteic metabolite released by dead and dying cells. Employing high-performance liquid chromatography (HPLC) analysis we measured significant amounts of ATP and inosine but not adenosine in protein-free supernatants (SNs) of irradiated melanoma cells. Assays with purified purinergic agonists and antagonists confirmed that inosine induces potent stimulation of tumor cell proliferation *via* adenosine receptors.

## Materials and Methods

### Reagents and Media

Dulbecco’s Modified Eagle’s Medium (DMEM), Roswell Park Memorial Institute 1640 medium (RPMI 1640), fetal bovine serum (FBS), penicillin–streptomycin, and glutamine were purchased from Gibco (Thermo Fisher, Germany). Trypsin-EDTA solution, adenosine, inosine, AMP, ADP, and ATP, the A2b (alloxazine) and A3 adenosine receptors (VUF5574), and caffeine, a non-selective adenosine antagonist, were purchased from Sigma-Aldrich (Germany). The antagonists for A1 (DPCPX) and A2a (SCH-58261) adenosine receptors were purchased from Tocris, UK.

### Cell Lines and Culture Conditions

The C57Bl/6 mouse-derived melanoma cell line B16F10 was purchased from ATCC (#CRL-6475) and propagated in DMEM supplemented with 10% FBS and penicillin/streptomycin (D10) at 37°C in a 5% CO_2_ atmosphere. NIH/3T3 fibroblast cell line was purchased from ATCC (#CRL-1648) and cultured in RPMI 1640 supplemented with 10% FBS, streptomycin/penicillin, and glutamine (R10). Human synovial tissue samples were obtained from knee joints of patients with rheumatoid arthritis from the orthopedic rheumatology unit of the Waldkrankenhaus St. Marienin Erlangen. An informed consent was obtained from patients, and their use was approved by the local ethics committee (Permit # 52_14B_3). Human fibroblast-like synoviocytes (FLS) were dissected by cutting off the villi of the synovial membrane. The tissue was digested using Collagenase IV solution (Sigma) in a shaking thermomixer at 37°C in two steps for 45 min. The samples were vortexed vigorously to release the cells. The collected cells were allowed to adhere to culture flasks for 2 days, with addition of fresh medium every day. Then, complete medium was removed together with non-attached cells, and cells were washed rigorously. Adherent cells were a mixture of two major cell subtypes: type A macrophage-like and type B FLS. Short trypsinization steps of about 2 min at each passage allow detachment of only fibroblastic-like cells and thereby removal of the monocytic cells from the cell mixture. The terminally differentiated macrophages have a limited life span *in vitro*, which contributes to the formation of a relatively clean pool of FLS. The isolated FLS were expanded in culture flasks for three passages by splitting them in a 1:2 ratio at confluency. The cells were used for proliferation assays in passages 3–6 in R10 medium supplemented with 1% fungizone (Gibco). Viable cells were seeded in 24-well plates at very low densities (200–1,000 cells/well) in the presence of dead and dying cells or their SNs. Control wells containing only 200 B16F10 cells in D10, 1,000 FLS in R10, or in indicated SN were included in the same plate during one experiment. Agonists, antagonists, and vehicle were added as indicated. Transwell experiments were performed with ThinCerts™ cell culture inserts from Greiner Bio-One employing a 0.4 µm pore size membrane on 24-well plates (Greiner Bio-One, Germany).

### Cell Death Induction and SN Preparation

Cell death was induced by irradiation with ultraviolet light type B (UVB) at 1.5 mJ/cm^2^/s or by heat shock (56°C for 30 min). The phenotype of cells after cell death induction was confirmed by a morphophysiological classification by flow cytometry (25) as previously shown [see Figures 2E,F in Ref. (13)]. At the indicated time points, SNs were collected, centrifuged at 6,000 *g* for 10 min, stored at −70°C until further use, and thawed only once. When necessary, the SNs were boiled on water bath for 15 min for deproteinization. A further protein-free fraction was obtained by filtration through Amicon^®^ (Millipore, Germany) filters with a 3 kDa size cutoff membrane.

### HPLC and Mass Spectrometry

High-performance liquid chromatography and size exclusion chromatography (HPLC–SEC) were performed with a Perkin Elmer Series 200 HPLC system using Strong Cation Exchange (SCX) column purchased from Shiseido CAPCELL PAK SCX UG 5 µm 150 mm × 1.5 mm. The protocol for detection of adenosine was developed based on manufacturer’s recommendations. The data was recorded for 10 pps and at 254 nm on column data with a 50 mM potassium dihydrogen orthophosphate (KH_2_PO_4_)–dipotassium hydrogen orthophosphate (K_2_HPO_4_) buffer (pH = 2.6) as a mobile phase and a flow rate of 0.5 ml/min (~860 psi) detection (26). The quantification of the concentration of purinergic metabolites was performed by liquid chromatography coupled with mass spectrometry.

### Measurement of Cell Proliferation

Cell cultures were harvested at the indicated time points by collecting the medium containing dead and spontaneously detached cell together with adherent cells after treatment with trypsin-EDTA solution for 20 min. Cell growth was quantified at different time points by flow cytometry employing a Gallios flow cytometer (Beckman-Coulter, Miami, FL, USA). Only viable cells excluding propidium iodide were recorded. Since the harvesting procedure required prolonged incubation and pipetting steps, an alternative colorimetric method for the quantification of growth was established for experiment requiring multiple simultaneous harvesting (27). Briefly, dead and non-adherent cells were washed out with warm PBS; adherent cells were fixed for 30 min in a solution of glutaraldehyde (1% in PBS), washed with PBS (pH 7.4), and subsequently stained with a 0.01% crystal violet solution (in 20% methanol). After removing excess of dye, the crystal violet-stained cells were dissolved in 1 ml of a 10% sodium dodecyl sulfate solution, and the optical density of the extracted dye was read in a spectrophotometer at 590 nm (27).

### *In Vivo* Tumor Cell Growth

Wild-type C57Bl/6 mice were bred at the Institute of Cell Biology, Lviv, Ukraine and kept on a standard diet with drinking water available *ad libitum*. A total of 5 × 10^5^ viable cells in Ringer’s solution, with adenosine or with SNs of dying cells (four mice per group), were implanted subcutaneously on the back of each mouse. Tumor volume was measured using a caliper from the day of appearance until the end of the experiment. Animal studies were conducted according to European principles and local guidelines for the care and use of laboratory animals. Experiments were performed on mice matched for age and sex and evaluated with blinded identity.

### Statistical Analysis

Data are reported as mean ± standard error of replicate mean values. The software package GraphPad Prism 5.0 was used for graphics and statistical tests. For comparisons between control and experimental groups, Mann–Whitney *U* test or two-way ANOVA tests were employed as appropriate. Statistical significance was assumed if *p* < 0.05.

## Results

### Stimulation of Cell Proliferation by Bystander Dead Cells, the “Feeder Cells” Effect

**Figure 1:**
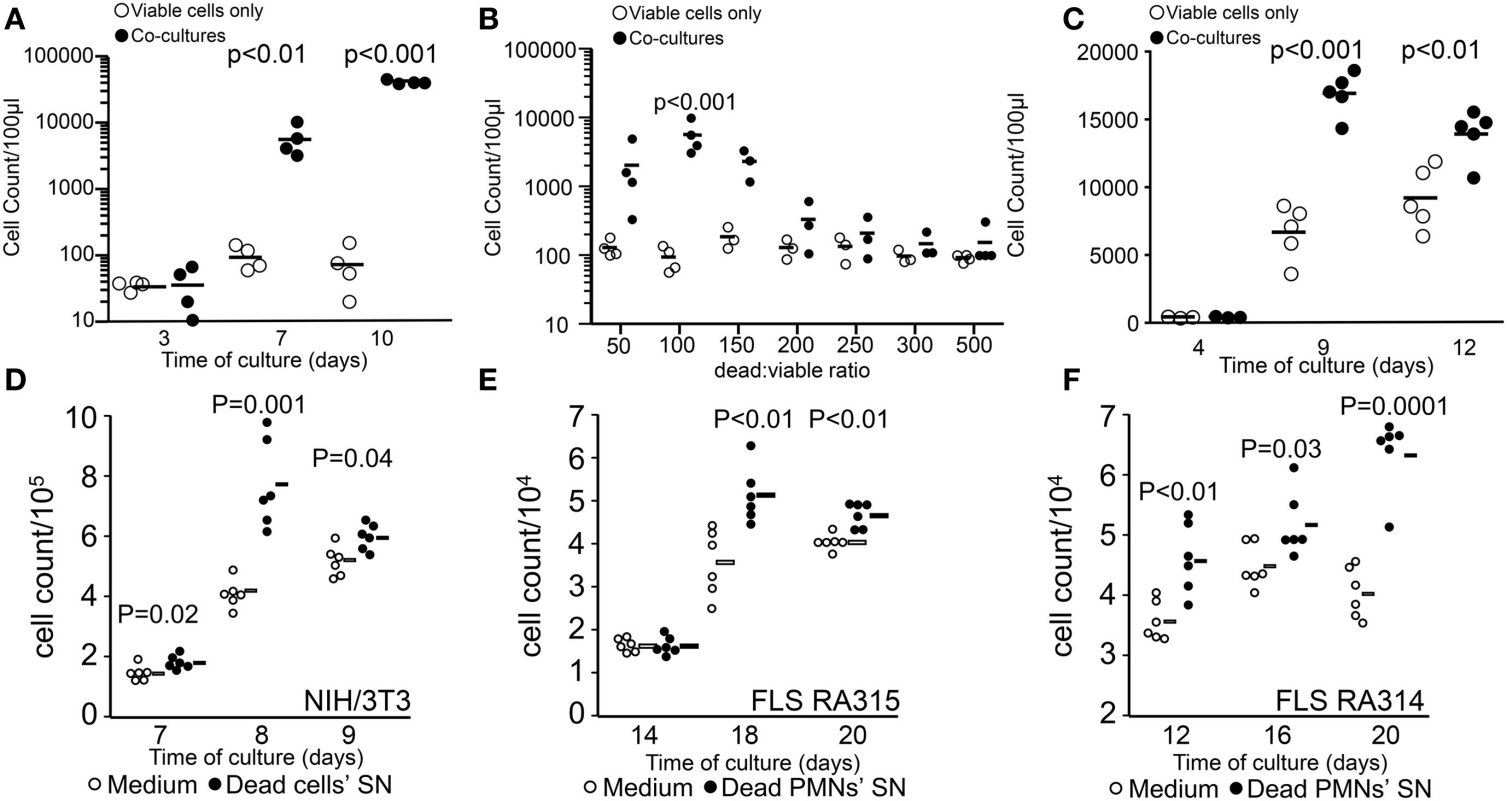
**Stimulation of tumor cell proliferation by bystander dying cells, the “feeder cells” effect**. The upper panel shows viable cell counts of cocultures [closed circles in panels **(A–C)**] and viable cells alone in D10 medium [open circles in panels **(A–C)**]. Cocultures were composed of viable (200 cells/well) and lethally ultraviolet light type B-irradiated (10,000 cells/well) B16F10 melanoma cells **(A)**. Dead/viable ratio titration of cocultures **(B)**. Transwell cocultures with 10,000 apoptotic cells **(C)**. The lower panel shows cell counts of independent wells containing fibroblasts in R10 medium [open circles in panels **(D–F)**] or in supernatants (SN) from apoptotic cells [closed circles in panels **(D–F)**]. Mouse NIH/3T3 fibroblast (1,000 cells/well) cultures with SNs of 0.2 million cells/ml apoptotic homologous cells **(D)**. Human fibroblast-like synoviocytes (FLS, 1,000 cells/well) from two rheumatoid arthritis patients RA315 **(E)** and RA314 **(F)** cocultured with SNs from apoptotic neutrophils (0.2 million cells/ml).

In order to simulate the conditions during cancer treatment where the vast majority of tumor cells are killed by irradiation and only a few survive, we evaluated the growth of few viable B16F10 cells (200 cells/well) in the presence of higher quantities of dying cells killed by UVB irradiation (apoptosis induction). Cultures were kept without adding fresh medium during the whole indicated culture period. Dying cells alone and viable cells alone were cultured simultaneously in separate wells as controls. We observed that cocultures of viable cells with dying cells by apoptosis supported rapid growth of viable cells seeded at very low densities. Seeding cells at these densities in D10 medium only showed no proliferation during the same period of time (Figure 1A). This effect was more pronounced when lower numbers of dying cells reach a maximum at the viable/dead cell ratio of 1:100 (Figure 1B). When the number of dead cells was further increased, the promotion of proliferation was completely abolished (Figure 1B). In order to explore whether physical contact between viable and dying cells is necessary to trigger cell growth enhancement, viable B16F10 cells were cultured in transwell plates (ThinCert Greiner, Germany), which allow physical separation of cells by a 0.4 µm pore membrane. Viable cells were plated in the upper side of the transwell, and 100 times more dying cells were placed in the lower compartment. The promotion of proliferation was also achieved when dying cells were separated from the viable ones (Figure 1C), indicating that soluble stimulating factors released by dead and dying cells concur to promote melanoma cell proliferation.

In order to elucidate whether the promotion of proliferation was specific for the melanoma cell line, we also employed NIH/3T3 mouse fibroblasts cultured with SNs from apoptotic homologous cells. Employing the staining method for adherent cells we quantified proliferation more efficiently as with the flow cytometric method and observed that the conditioned medium from irradiated fibroblast significantly stimulated the proliferation compared to fibroblasts cultured in R10 medium alone (Figure 1D). Furthermore, the source of the stimulatory potential and the proliferating target cell were not limited to immortalized cells. Primary FLS from patients with rheumatoid arthritis were also stimulated to proliferate by SNs of apoptotic neutrophils (Figures 1E,F).

### Dying and Dead Cells Release Proliferation-Stimulating Factors

**Figure 2:**
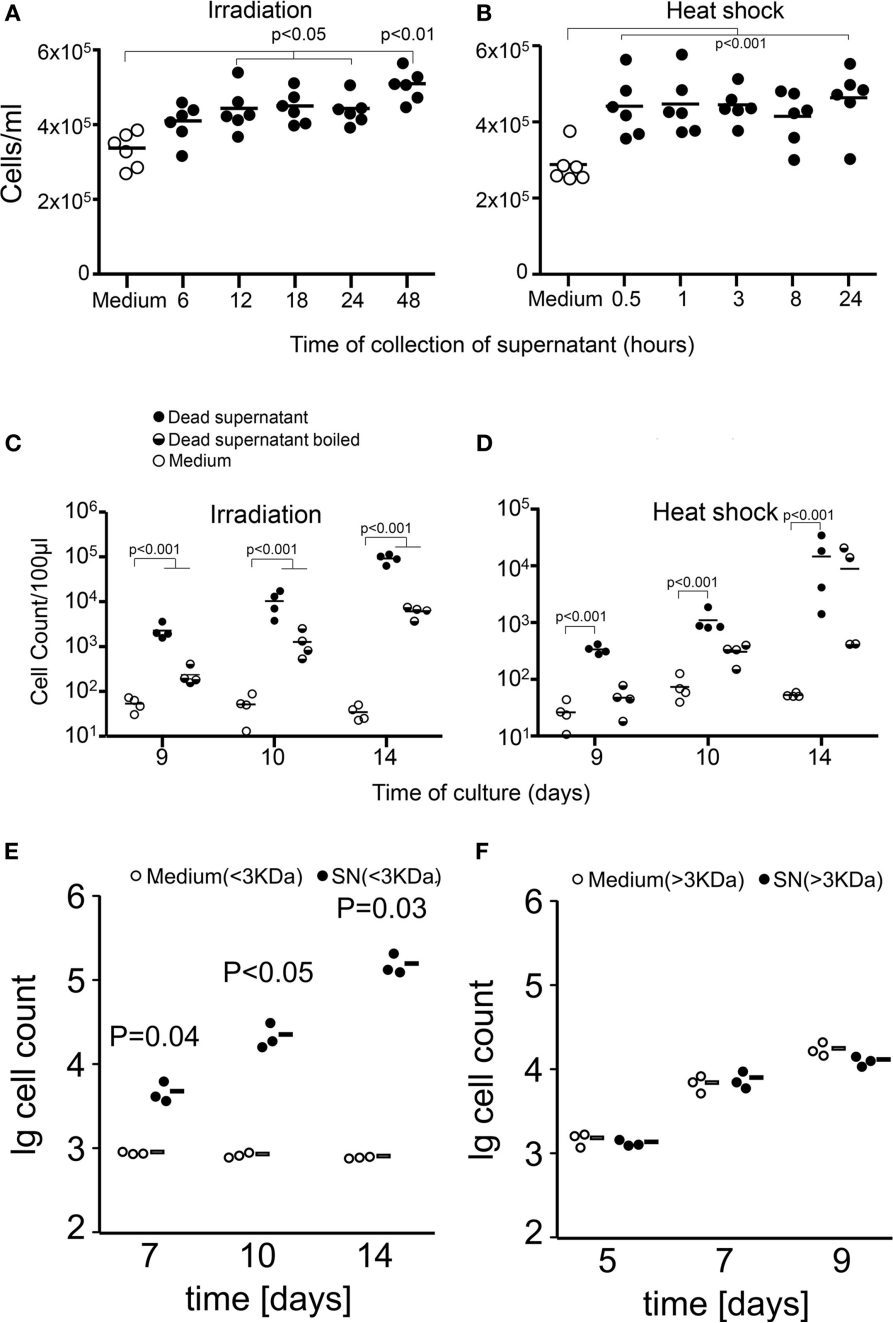
**Apoptotic and necrotic cells release proliferation-stimulating factors**. Shown are cell counts of cultures of viable cells in medium alone (open circles) or in supernatants (SNs) from dead and dying cells (closed circles). Factors stimulating proliferation are released by dying and dead cells after 12 h of irradiation [apoptosis **(A)**] and immediately after heat shock [necrosis **(B)**]. Proliferation was measured by the crystal violet assay for adherent cells. Boiling of SNs (half-full circles) partially reduces but not abolishes the stimulation of proliferation of tumor cells [24 h after irradiation **(C)** and 1 h after heat shock **(D)**]. Factors stimulating the proliferation of tumor cells are present in the protein-free fraction **(E)** and not in the protein-rich fraction **(F)** of dead cell SNs.

The analysis of SNs collected at different time points after the induction of cell death showed that cells dying by apoptosis after irradiation released the stimulating factors after 12 h of culture and lasted for 36 h more (Figure 2A). In contrast, heat shock treatment resulted in immediate necrosis and release of proliferation-stimulating factors (Figure 2B). In order to investigate the nature of the released factors, we boiled SN collected after 24 h of irradiation and after 8 h of heat shock treatment and compared the stimulating capacity with that of untreated SNs and fresh D10 medium. We found that boiling only partially reduced the stimulating capacity of the SNs from both apoptotic (Figure 2C) and necrotic cells (Figure 2D). These results suggest that proteins released after cell death are unlikely to be responsible for the stimulation of proliferation in melanoma cells. We confirmed this hypothesis when we prepared the protein-free and -rich fractions by filtration through a 3 kDa membrane of SNs and medium. We observed that the proliferation-stimulating activity was present only in the protein-free fraction of dead cell SNs (Figures 2E,F). Interestingly, cells proliferated even in the absence of high molecular weight nutrients, which rather represents a starving condition.

### Molecular Analysis of the Protein-Free Fraction of SNs from Dying Cells

**Figure 3:**
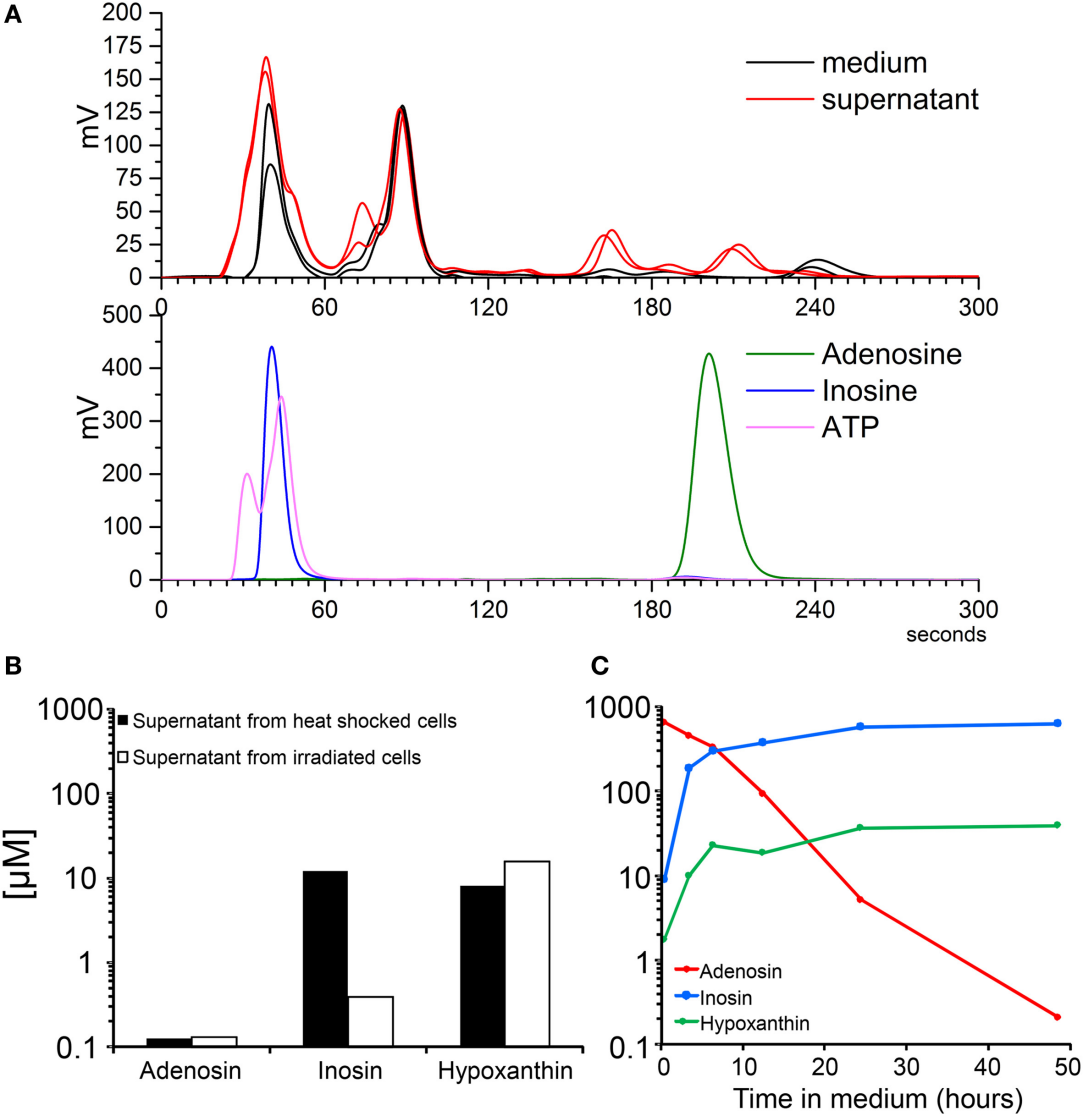
**Molecular analysis of the protein-free fraction of supernatants (SNs) from dying cells**. Electrograms of SNs of B16F10 cells after 24 h of irradiation and metabolite standards by high-performance liquid chromatography **(A)**. Concentrations of nucleosides measured by mass spectrometry of SNs of B16F10 cells after 24 h of irradiation and 1 h after heat shock **(B)**. Spontaneous adenosine degradation into its metabolites inosine and hypoxanthine in SN spiked with 500 µM of adenosine and measured by mass spectrometry **(C)**.

One of the most important non-proteic metabolites released is the nucleotide ATP. Therefore, we set up a HPLC–SEC analysis of the composition of protein-free fractions of SNs from dying cells contrasted with the standards ATP, adenosine, and inosine. The runs for two samples of SNs and fresh medium are shown in Figure 3A, upper panel. The purified standards are shown in the lower panel (Figure 3A). The chromatographic separation of the metabolites allows speculating about ATP and inosine being present in the SNs of dying cells. The same is not true for adenosine. The mass spectrometric analysis confirmed the presence of inosine (10 nM) or its metabolite hypoxanthine (10 nM) in SNs of apoptotic and necrotic cells, respectively (Figure 3B). Adenosine was detectable but at much lower concentration (<0.2 μM; Figure 3B). Adenosine in the body is produced by the action of ectonucleotidases from nucleotides like ATP, ADP, and AMP. Adenosine is either immediately transported to the intracellular space or is degraded by the action of adenosine deaminase into inosine (28). The phosphorylation of the ribose from the inosine results in the generation of hypoxanthine and ribose-1-PO_4_ (29). We confirmed that all these steps of the purinergic metabolism take place spontaneously in cell-free SNs. The addition of 500 µM purified adenosine to cell-free SNs from dying cells resulted in the clearance of adenosine and the production of inosine and hypoxanthine (Figure 3C). These results confirm that nucleotides are released by dying cells and are immediately catabolized by ectonucleotidases present in the culture into adenosine and inosine.

### Adenosine Receptors Mediate the Stimulation of the Proliferation Induced by SNs of Dead and Dying Cells

**Figure 4:**
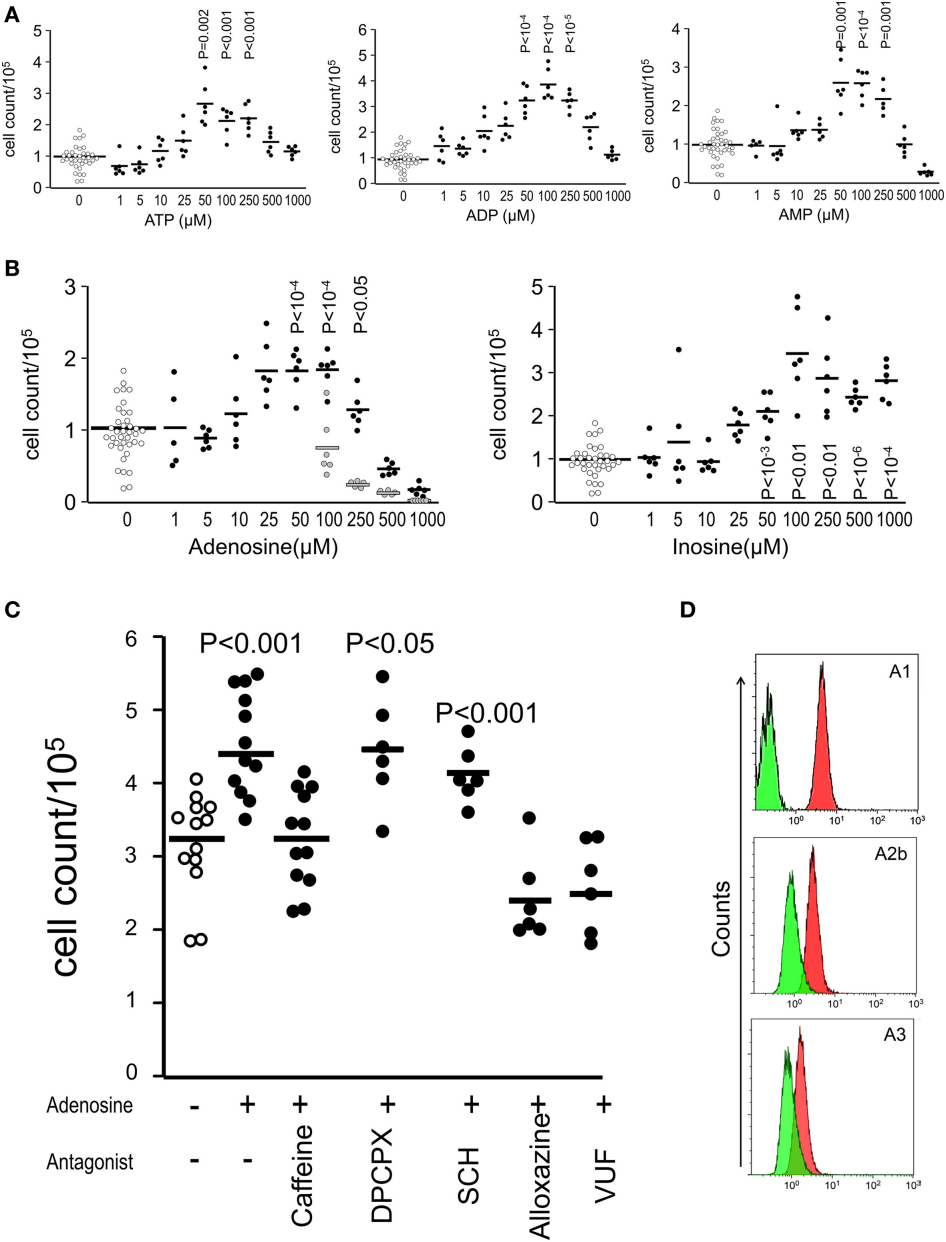
**Adenosine receptors mediate the stimulation of the proliferation induced by supernatants of dead and dying cells**. Shown are cell counts of cultures measured by the crystal violet method for adherent cells at day 9 showing the relative potency of nucleotides **(A)** and nucleosides **(B)** on the stimulation of proliferation of B16F10 melanoma cells (D10 medium wells are open circles, purinergic metabolites are closed circles, and vehicles for adenosine are gray circles). The pan-adenosine receptor antagonist caffeine and the specific adenosine receptor antagonists alloxazine (A2bR) and VUF (A3R) blocked the stimulation of proliferation induced by inosine **(C)**. Adenosine receptor expression on B16F10 cells **(D)**. Green represent the isotype antibody binding and red the receptor antibody.

After confirming the presence of inosine and ATP as major purinergic metabolites in the SN of both apoptotic and necrotic cells, we investigated the relative potency of the purified nucleotides ATP, ADP, and AMP (Figure 4A) and nucleosides adenosine and inosine (Figure 4B) after 9 days of culture of melanoma cells without feeding. Nucleotides begin to exert stimulating activity at 10 µM concentrations (Figure 4A), whereas nucleosides stimulate proliferation at 50 µM concentration (Figure 4B). Both types of metabolites exhibited a bell-shaped dose–response curve similar to that observed for dead/viable cells cocultures (Figure 1B).

In order to investigate whether adenosine receptors participate in the stimulation of proliferation induced by purinergic metabolites, we added the pan-adenosine receptor antagonist caffeine and the specific adenosine receptor antagonists alloxazine (A2bR) and VUF (A3R) to adenosine-stimulated cultures and observed that the stimulation of proliferation most likely mediated by inosine was abolished in the presence of the antagonists for the adenosine receptors A2b and A3 and not by the antagonists of the receptors A1 and A2a (Figure 4C). Additionally, we confirmed the expression of the adenosine receptors A1, A2b, and A3 on the surface of viable B16F10 melanoma cells (Figure 4D).

### Stimulation of Proliferation *In Vivo*

**Figure 5:**
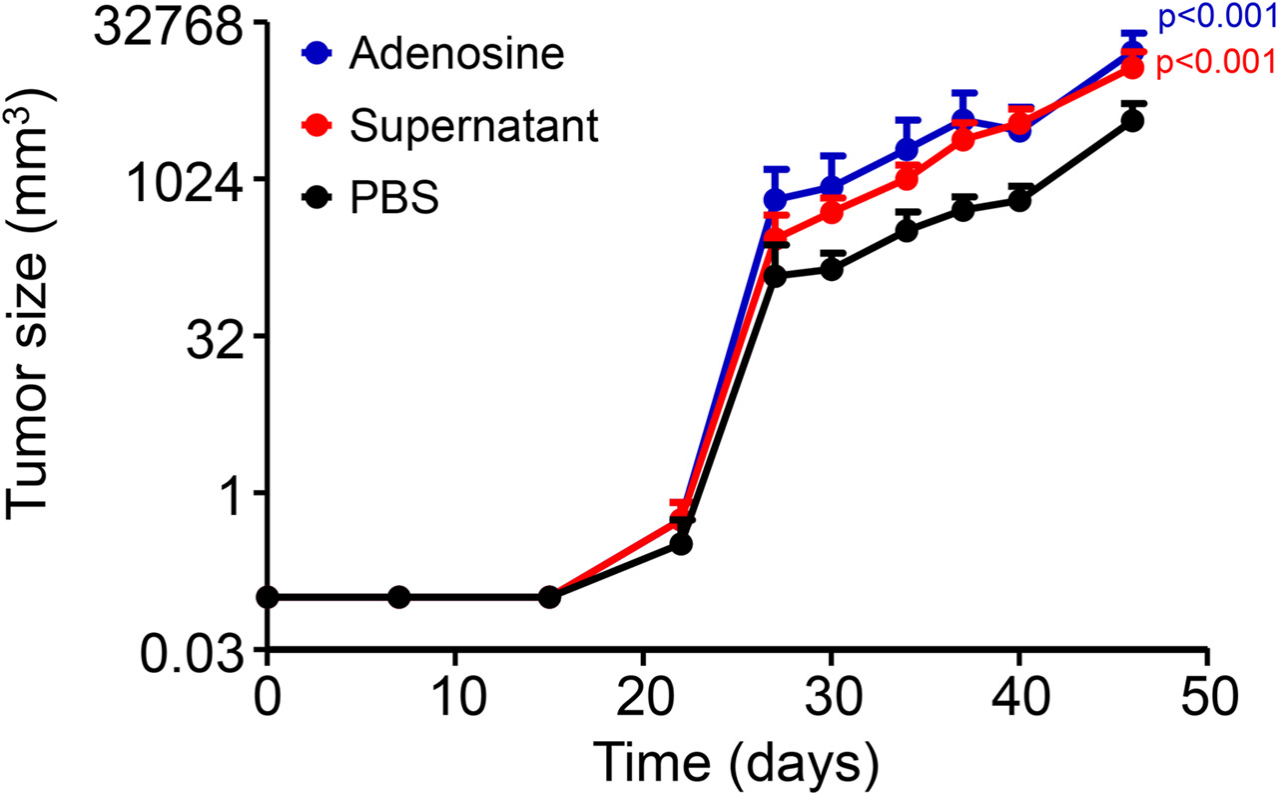
**Stimulation of proliferation *in vivo***. The presence of adenosine or supernatant of irradiated cells at the site of implantation of 50,000 B16F10 cells caused development of larger subcutaneous tumors in C57Bl/6 mice (*n* = 6 per group).

The implantation of few (50,000) B16F10 cells in the subcutaneous space of the back of C5Bl/6 mice led to the late development of solid tumors. The first measurable tumor appeared at day 24 after implantation (Figure 5, black line). Coimplantation with either SNs from apoptotic cells or with 200 µM adenosine caused the development of larger tumors (Figure 5, colored lines).

## Discussion

During tissue turn over, dying cells are swiftly cleared by innate immune sentinels in a silent manner (30). Many conditions of health and disease are clearly modulated by the presence and effects of apoptotic cells (31). The induction of tumor cell death is a central goal of chemo- and radiotherapy. Therefore, the understanding of modulatory signals from dying and dead cells might be important for clarifying some undesirable outcomes after cancer therapy (32, 33). The first observation about signals derived from dying cells supporting either cell proliferation or tumor growth came from early works of Revesz in 1956 on mammary carcinoma C3H cells killed by X-rays (14). By that time, it was referred as “a feeder effect in which the dead cells released essential nutrients.” Currently, it is well known that apoptotic cells possess regenerative properties. A potentially important aspect of the called “feeder effect” of apoptotic cells in the tumor microenvironment is their capacity to activate compensatory proliferative responses in their neighborhood (34). The basis for this potential lies in responses to wounding, notably in *Drosophila, Xenopus*, and *Hydra*. The molecular mechanisms underlying the proliferative responses elicited by dying/dead cells are far from clear and include Wnt and MAPK signaling (35). We have previously reported that the growth-promoting factors are released by apoptotic B16F10 melanoma cells and acted at remote sites in an *in vivo* model of tumor growth (18). However, all these findings only partially explained the observed regeneration phenomena and were limited to irradiated cells or wound healing (15–17). In this work, we describe the particular features of proliferation-promoting factors that lead us to identify inosine as a putative main mediator of tumor repopulation after massive death of tumor cells.

One of the most important features of the proliferation-supporting activity is its restriction to a range of concentrations. High and very low densities of dying/dead cells did not stimulate cell proliferation *in vitro*. Adverse conditions are known to be present in SNs of dying/dead cells (36) and may explain why high densities of dying cells have deleterious effects for cell proliferation. The induction of proliferation of viable cells also occurred when dead ones were physically separated, confirming the soluble nature of the factor derived from dead cells. This observation supports previous findings, where dying cells implanted at distant location from viable also promoted the growth of tumors *in vivo* (18). Finally, the fractionation of SN of necrotic cells resulted in stimulation of proliferation in conditions that otherwise resulted in death by starvation. Based on these observations, we propose here for the first time that both apoptotic and necrotic cells promote the stimulation of proliferation of viable cells mainly through the release of soluble factors. This is in line with the idea of a highly conserved homeostatic mechanism driven by dead/dying cells to maintain the homeostasis of an organ.

In order to further characterize the nature of the stimulating signal, we killed cells with increasing temperatures and observed that the effect persists at 70°C (data not shown). Furthermore, SNs of dying cells boiled at 100°C for 15 min were still able to support cell proliferation of viable cells. These observations clearly support the involvement of non-proteic bioactive molecules in the stimulation of proliferation released from dead and dying cells. The most important chemotactic mediators released by apoptotic cells are ATP (7) and adenosine (12). Therefore, we narrowed the molecular analysis of the SNs by HPLC–SEC and mass spectrometry to purinergic metabolites. We found traces of ATP and inosine but not adenosine in the SNs of apoptotic cells. Since ectonucleotidases and adenosine deaminase are broadly expressed in neoplastic and normal cells, we assume that released ATP can be rapidly transformed to ADP, AMP, adenosine, and inosine in the SN. We confirmed this assumption by adding adenosine to an apoptotic cell SN and incubating for 48 h. The transformation of adenosine to inosine suggests the presence of active adenosine deaminase in the SNs of apoptotic cells.

The concentrations of inosine or hypoxanthine measured in the SNs of apoptotic and necrotic cells roughly reflect the minimal amount of purified inosine required to induce proliferation in melanoma cells. Adenosine has reportedly growth-promoting and growth-inhibitory effects depending on the tumor type (37–39). We could only verify the stimulating capacity of adenosine in our system, and it seems to be limited by the concentration of the adenosine vehicle ammonia. It has already been reported that inosine, and not adenosine, exerts potent proliferation-stimulatory actions on melanoma cells, mainly through the engagement of A3 adenosine receptor (40). Since we have not inhibited the catabolism of adenosine to inosine in the SNs, it is likely that the stimulation of proliferation of melanoma cells is ultimately supported by inosine. The specific inhibition of adenosine receptors on melanoma cells further confirms that inosine is an important stimulator of cell proliferation and may act as a mediator of the healing and repopulation processes subsequent to massive cellular damage.

The generation of larger tumors *in vivo* during the same time frame and starting from the same amount of regenerating cells supports the hypothesis that the presence of purinergic metabolites in the micromilieu surrounding surviving tumor cells plays a decisive role in the outcome of the applied therapy, especially if cell death has been massively induced. Concentrations of nucleosides in the microenvironment of solid tumors have been rarely measured. In solid carcinomas, the concentrations of adenosine ranged from 0.2 to 2.4 μM and were significantly higher than healthy tissues (22). Interestingly, various types of cancers express adenosine receptors, for example, A1, A2a, and A3 adenosine receptors have been found in human colorectal carcinoma (41–43), human leukemia Jurkat T cells (44), F98 glioblastoma cells (45–47), and melanoma cells (48, 49). The adenosine receptor A2b has also been found in melanoma cells (50) and is strongly associated with neovascularization of tumors. Our results provide a novel link between induction of cell death and the release of potent stimulators of cell proliferation that can be exploited by surviving cells. A systematic measurement of nucleosides (51), especially inosine, in the microenvironment of solid tumors in combination with tumor progression data may contribute to improve the prognosis of cancer patients. Therapeutic interventions leading to cause tumor cell death may take advantage of the enormous progress been made in the pharmacology of adenosine receptor targeting (23).

## Ethics Statement

Animal experiments were conducted with bioethical permission from the Bioethics committee Danylo Halytsky Lviv National Medical University, Protocol 6, dated June 24, 2013. Experiments with human material were approved by the ethics committee of the Friedrich-Alexander University (Permit # 52_14B_3).

## Author Contributions

JC, RC, LM, and CM planned and performed most of the *in vitro* and *in vivo* experiments, conducted data analysis, and wrote the manuscript. MR and RB performed the molecular analysis of supernatants. AD and AH provided patient material, methodology and provided scientific input. MH, YL, XM, and YZ provided scientific input and wrote the manuscript. MH, LM, and YZ supervised the project, planned, and conducted experiments, data analysis, and wrote the manuscript. All the authors read and approved the manuscript.

## Conflict of Interest Statement

The authors declare that the research was conducted in the absence of any commercial or financial relationships that could be construed as a potential conflict of interest.
